# Construction and Characterization of Single-Chain Variable Fragment Antibody Library Derived from Germline Rearranged Immunoglobulin Variable Genes

**DOI:** 10.1371/journal.pone.0027406

**Published:** 2011-11-11

**Authors:** Man Cheng, Shirley Y. W. Chan, Qi Zhao, Elaine Y. M. Chan, Shannon W. N. Au, Susanna S. T. Lee, Wing-Tai Cheung

**Affiliations:** 1 School of Biomedical Sciences, Chinese University of Hong Kong, Hong Kong, China; 2 School of Life Sciences, Chinese University of Hong Kong, Hong Kong, China; University of Rome, Italy

## Abstract

Antibody repertoires for library construction are conventionally harvested from mRNAs of immune cells. To examine whether germline rearranged immunoglobulin (Ig) variable region genes could be used as source of antibody repertoire, an immunized phage-displayed scFv library was prepared using splenocytic genomic DNA as template. In addition, a novel frame-shifting PCR (fsPCR) step was introduced to rescue stop codon and to enhance diversity of the complementarity-determining region 3 (CDR3). The germline scFv library was initially characterized against the hapten antigen phenyloxazolone (phOx). Sequence analysis of the phOx-selective scFvs indicated that the CDRs consisted of novel as well as conserved motifs. In order to illustrate that the diversity of CDR3 was increased by the fsPCR step, a second scFv library was constructed using a single scFv clone L3G7C as a template. Despite showing similar binding characteristics towards phOx, the scFv clones that were obtained from the L3G7C-derived antibody library gave a lower non-specific binding than that of the parental L3G7C clone. To determine whether germline library represented the endogenous immune status, specific scFv clones for nucleocapsid (N) protein of SARS-associated coronavirus (SCoV) were obtained both from naïve and immunized germline scFv libraries. Both libraries yielded specific anti-N scFvs that exhibited similar binding characteristics towards recombinant N protein, except the immunized library gave a larger number of specific anti-N scFv, and clones with identical nucleotide sequences were found. In conclusion, highly diversified antibody library can be efficiently constructed using germline rearranged immunoglobulin variable genes as source of antibody repertoires and fsPCR to diversify the CDR3.

## Introduction

Phage-displayed antibody library has been widely used to derive high-affinity target-specific antibodies, such as antibodies that were specific for angiogenesis marker fibronectin [1], melanoma-specific B3 and B4 antigens [2], epidermal growth factor receptor [3], HIV Vpr protein [4], and spike protein of SCoV [5], [6]. Antibody repertoires of phage-displayed library are conventionally created by harvesting mRNAs from peripheral blood lymphocytes, spleen, bone marrow, tonsil or similar sources using RT-PCR and family-based oligonucleotides [7], [8], [9], [10]. The heavy and light chains are then randomly combined and cloned to construct a combinatorial scFv library, from which specific antibodies against not-yet-encountered antigen are selected [11], [12], [13], [14].

Although the use of mRNAs ensures functional antibody genes retrieval, there are some limitations. Potential Ig genes may not be recovered from antibody cDNA library due to reading frame shifted or the presence of stop codon(s) which are generated by imprecise somatic recombination and P- and N- additions [15], [16]; or the immunoglobulins are self-reactive and thus eliminated by the host immune system [17], [18]. Besides, like other somatic cells, B cells are diploid and therefore rearranged Ig genes can only be expressed from one of the sister chromosomes while the other is concealed [18], [19]. In addition, chance of getting Ig genes against poor immunogenic targets is hampered by poor humoral response of immunized host. On the other hand, activated B cells undergo clonal expansion and therefore the antibody repertoires of a cDNA-derived scFv library would be dominated by antigen-stimulated humoral response. Hence recombinant antibody repertoires of a cDNA-derived antibody library are limited.

Germline Ig variable region (V) genes, which are selected over millions of years for their compatibility with many different antigens, are poly-functional and capable of orchestrating an effective immune-response [20], [21]. Indeed, antibodies that encoded by a very limited V_H_ and V_L_ genes of inbred mice were found to react with different haptens, polysaccharides, and even protein antigens [22], [23], [24], [25], [26]. However, the potential use of rearranged germline Ig genes as source of antibody repertoires for construction of antibody library has never been explored.

Structural analysis of antibody binding site suggests that only a few canonical conformations exist within the five CDRs except V_H_ CDR3 loop which shows a wide range of variations in both length and space [27], [28]. Previous studies indicate that CDR3 diversity is the principle determinant of antigen-specificity and binding-affinity [29], [30], and the diversity of CDR3-FR4 junction determines how antibody undergoes affinity maturation [31]. Hence, modifications in CDR3 seem to be an efficient way of expanding antibody diversity beyond what are encoded by the germline Ig genes.

In the present study, we described the construction and characterization of germline scFv antibody fragment libraries using variable region of rearranged Ig genes as source of antibody repertoires. The germline Ig variable regions were further diversified using a novel frame-shifting PCR step to rescue stop codon(s) as well as to enhance diversity in the CDR3 loop. Indeed, in this proof-of-concept study, we showed that the germline scFv library was highly diverse, and specific antibodies against hapten and protein were obtained.

## Materials and Methods

### Oligos and primers

All oligos and primers were custom synthesized by Invitrogen. The sequences and the relative position with respect to an scFv of various oligos and primers were detailed in Table 1 and Figure S1, respectively.

**Table 1:** Primer Pairs for Construction of Germline Phage-displayed scFv Library.

***A. Mouse V_H_ Forward Primers (FR1 Region)***	
FH1	5′- gAggTgMWgcTTRT-3′
FH2	5′-gAggTgMWgcTKVWgSAgTcTggA-3′
***B. Mouse V_H_ Reverse Primers (FR4 Region)***	
RH1	5′- gAcDgTgASHGWRGTYC -3′
RH2	5′-gAcDgTgASHRDRgTBccTKSRccccA-3′
***C. Mouse V_Lκ_ Forward Primers (FR1 Region)***	
FK11	5′-gAHRTYgTKMTSAc-3′
FK12	5′-gAHRTYgTKMTSAcMcARWcTMcA-3′
***D. Mouse V_Lκ_ Reverse Primers (FR4 Region)***	
RK11	5′-KATYTccARYYTKgT-3′
RK12	5′-KATYTccARYYTKgTSccHBcDccgAA-3′
***E. Frame-shifting primers***	
RFSH	5′-gAcDgTgASHRDRgTBccTKSRccccANNNNNN-3′
RFSK	5′-YYTKgTSccHBcDccgAAYgTNNNNNN-3′
***F. Linker primers for V_H_***	
SBS1	5′-cgAgcTcggATccggcccAgccggccSAggTgMWgcTKVWgSAg-3′
L1JP	5′-AgAAccgcTgccTgAAccgccTccAccAcTgAcDgTgASHRDRgTBccT-3′
***G. Linker primers for V_Lκ_***	
L2JP	5′-cAggcAgcggTTcTAgcggcggTggcggAgAHRTYgTKMTSAcMcARWc-3′
KN1	5′-cggggTAccgcggccgcKATYTccARYYTKgTSccHBcDccgAA-3′

For the primer nucleotide sequence, the non-standard bases are: N = A+G+C+T; R = A+G; Y = C+T; M = A+C; K = G+T; S = G+C; W = A+T; H = A+C+T; B = G+C+T; D = A+G+T; V = A+G+C.

### Isolation of splenocytes

The protocol for animal work was approved by the Animal Experimentation Ethics Committee of the Chinese University of Hong Kong (Permit Number: 03/015/MIS). BALB/c mice (∼25 g) were sacrificed by cervical dislocation and the spleens were immediately removed. After punching holes with a G21 needle, splenocytes were squeezed out and resuspended in an ice-cold phosphate buffered saline (PBS) containing 4% BSA (Sigma). For isolation of CD19^+^ cells, splenocytes (2×10^7^ cells/ml, 5 ml) were incubated with a fluorescein isothiocyanate (FITC)-conjugated rat anti-mouse CD19^+^ monoclonal antibody (0.5 mg/ml, 100 µl, BD Bioscience) on ice for 30 min to label B-lymphocytes carrying rearranged immunoglobulin genes. After washing to remove unbound antibodies, labeled cells were resuspended in 5 ml of RPMI-1640 medium (Gibco) supplemented with 10% fetal bovine serum (FBS, Gibco). The CD19^+^ splenocytes were sorted and captured using a FACS VANTAGE SE cell sorter (Becton Dickinson).

### Retrieval of variable regions from germline rearranged Ig genes

Splenocytic genome DNA was extracted using DNAzol® reagent (Invitrogen) as described by the manufacture. The variable regions of germline rearranged Ig genes were then retrieved using semi-nested PCR (snPCR). The 1^st^ round snPCR aimed at amplifying the rearranged Ig variable regions using genome DNA as template, and reaction was carried out in a final volume of 50 µl containing 1X PCR buffer (Perkin-Elmer), 1.5 mM MgCl_2_ (Perkin-Elmer), 0.2 mM dNTP (Invitrogen), 5% DMSO (MB grade from Sigma), 2.5 U non-proof reading Taq polymerase, 200 ng of extracted splenocytic genome DNA and V_H_ degenerate primer pair of 0.3 µM FH1 and 1.5 µM RH1, or V_Lκ_ degenerate primer pair of 0.45 µM FK11 and 0.3 µM RK11. Following pre-denatured at 94°C for 60 s, the reaction mixture was subjected to PCR amplification that consisted of two stages. The first stage was composed of 10 amplification cycles each consisting of 4 amplification steps, including denaturation at 94°C for 30 s, annealing at 40°C for 60 s, a ramping up extension step from 40°C to 72°C at a rate of 0.2°C/s, and then followed by an extended incubation step at 72°C for 1 min. Then the samples were subjected to another stage of 20 amplification cycles each consisting of denaturation at 94°C for 30 s, a touch up annealing step from 40°C to 50°C at a rate of 0.5°C/cycle for 1 min, and an extension step at 72°C for 60 s. Following the amplification cycles, the reaction mixture was further subjected to a post-extension step at 72°C for 2 min. The PCR reactions were carried out in a GeneAmp® 9700 PCR thermocycler with 96-well aluminum plate (Applied Biosystems). The PCR products were stored at 4°C until use.

To enrich Ig variable region fragments, PCR products of 1^st^ round snPCR were used as templates for 2^nd^ round of snPCR with primer pair carrying additional oligonucleotides at the 3′-end that bracketed only Ig-like templates being further amplified (Figure S1). The 2^nd^ round snPCR was carried out in a final volume of 50 µl containing 1X PCR buffer; 1.5 mM MgCl_2_, 0.2 mM dNTP, 5% DMSO, 2.5 U non-proof reading Taq polymerase, and 4 µl of the 1^st^ round PCR products. For retrieving V_H_ segments, 0.3 µM FH2 and 1.5 µM RH2 were used. For V_Lκ_ semi-nested PCR, 0.5 µM FK12 and 1.125 µM RK12 were used. Reaction mixture was pre-denatured at 94°C for 2 min, and then subjected to PCR amplification that consisted of three stages. The first stage is composed of 5 amplification cycles each consisting of denaturation at 94°C for 30 s, annealing at 65°C for 30 s and extension at 72°C for 30s. The second stage was composed of 25 amplification cycles each consisting of a denaturation step at 94°C for 30s, a touch-down annealing step from 65°C to 55°C at a rate of 0.4°C/cycle for 30 s and an extension step at 72°C for 30 s. The third stage was composed of 5 amplification cycles each consisting of a denaturation step at 94°C for 30s, an annealing step at 55°C for 30 s and an extension step at 72°C for 30 s. Following the amplification cycles, the reaction mixture was further subjected to a post-extension step at 72°C for 2 min. The PCR products were stored at 4°C until use.

To purify the Ig variable region gene fragments, PCR products were pooled and subjected to agarose gel electrophoresis. Agarose gel stripes containing the Ig gene fragments were placed into a dialysis tubing (MWCO 3,500 from Spectrum) together with 1 ml of TAE buffer, DNA fragments were electro-eluted, purified by phenol chloroform extraction and then precipitated by ethanol. The purified PCR products were either cloned into TOPO TA (Invitrogen) or pGEM-T Easy (Promega) cloning vectors for nucleotide sequence determination, or used for subsequent CDR3 diversity enhancement.

### Frame-shifting PCR for CDR3 diversity enhancement

The frame-shifting PCR (fsPCR) was performed as described in our US patent [32]. Briefly, the frame-shifting reaction was carried out in a final volume of 50 µl containing 1X PCR buffer, 1.5 mM MgCl_2_, 0.2 mM dNTP, 2.5 U non-proof reading Taq polymerase, 60-100 ng gel-extracted V_H_ or V_Lκ_ DNA fragments, and V_H_ primer pair of 0.5 µM FH1 and 4 µM V_H_ frame-shifting reverse primer RFSH; or V_Lκ_ primer pair of 0.5 µM FK12 and 4 µM V_Lκ_ frame-shifting reverse primer RFSK. For both V_H_ and V_Lκ_ fsPCR, the reaction mixture was pre-denatured at 94°C for 2 min. The 25 fsPCR cycles were carried out in a GeneAmp® 9700 PCR thermocycler with 96-well aluminum plate (Applied Biosystems). Each fsPCR cycle consisted of a denaturation step at 94°C for 30 s, an annealing step at 20°C for 2 min and an extension step with temperature ramping up from 20 to 94°C at a rate of +0.1°C per second (or 5% ramping-speed of GeneAmp® 9700 PCR thermocycler with 96-wells aluminum plate). The fsPCR products were stored at 4°C until use. After separation on a 1.5% agarose gel, DNA fragments with a size range of 300–400 bp were electro-eluted, phenol-chloroform purified, and ethanol precipitated. The purified PCR products were either cloned into TOPO TA (Invitrogen) or pGEM-T Easy (Promega) cloning vectors for nucleotide sequence determination, or used for subsequent scFv construction.

### ScFv construction

The CDR3-diversified germline Ig V_H_ and V_Lκ_ DNA fragments were randomly linked together using a two-stage overlap extension PCR (oePCR). The 1^st^-stage oePCR was carried out in a reaction volume of 50 µl containing 1X PCR buffer, 1.5 mM MgCl_2_, 0.2 mM dNTP, 2.5 U non-proof reading Taq polymerase, 120 ng of frame-shifted Ig V_H_ or V_Lκ_ DNA fragments, and 0.2 µM each SBS1 and L1JP (V_H_ primer pair); or L2JP and KN1 (V_Lκ_ primer pair). The 1^st^-stage oePCR was used to add an adaptor and a linker to the retrieved Ig variable region of heavy or light chains. After a pre-denaturing step at 94°C for 2 min, 15 amplification cycles were carried out. Each cycle consisted of a denaturation, an annealing, and an extension steps at 94°C, 54°C, and 72°C for 30 s, respectively. After an extended incubation at 72°C for 5 min, the PCR products were stored at 4°C until use. The PCR products (5 µl) were used as templates for the subsequent 2^nd^-stage oePCR for scFv construction.

The 2^nd^-stage of oePCR was used to join the heavy and light chains randomly into an scFv. The reaction was carried out in a reaction volume of 50 µl containing 1X PCR buffer, 1.5 mM MgCl_2_, 0.2 mM dNTP, 2.5 U non-proof reading Taq polymerase, 5 µl of PCR products of the 1^st^-stage oePCR (both V_H_ and V_Lκ_) and 0.2 µM each SBS1 and KN1 primers. The mixture was pre-denatured at 94°C for 2 min, then subjected to 20 amplification cycles. Each cycle consisted of denaturation at 94°C for 30 s, annealing at 62°C for 30s, and extension at 72°C for 90s. After an extended incubation at 72°C for 5 min, the PCR products were stored at 4°C until use.

### ScFv Library construction and specificity assessment

The Not 1- and Sfi 1- restricted scFv repertoires were cloned into pCANTAB 5E phagemid vector (GE Healthcare). Library was constructed by electroporation of the ligated products into competent TG1 *E. coli* as described in our previous publication [33]. Subsequently, log-phase TG1 transformants were super-infected with M13KO7 helper phage (GE Healthcare) in a multiplicity of infection (moi) ratio of 3∶1, and phages were rescued at 30°C overnight with gentle shaking. The overnight culture of scFv-phages was purified by polyethylene glycol precipitation (20% PEG8000 and 2.5 M NaCl). Purified phages were resuspended in 4 ml of a pre-blocking buffer (1X PBS, 0.2% Triton X-100, 0.01% NaN_3_, 0.1% BSA and 10% non-fat milk) and incubated at room temperature for 30 min prior panning. Phages (0.5 ml/well) were panned against immobilized antigen as described previously [34], [35]. Briefly, antigen (5 – 200 µg) was immobilized in a 24-well plate and incubated with the scFv library. After incubation at room temperature for 2 hr with gentle shaking, bound scFv-phages were eluted with 100 µl of 0.1 M glycine-HCl, pH 2.2. After 10 min acid-incubation at room temperature, the eluant was neutralized with 10 µl of 1 M Tris-HCl, pH 8.0. Specificity of eluted phages was confirmed by phageELISA and competitive phageELISA with free ligand in antigen-competition manner, bound phages were detected with an anti-M13 peroxidase-conjugated antibody (GE Healthcare).

### Nucleotide sequence analysis

Nucleotide sequence determinations were performed by dye-terminator cycle sequencing using Beckman CEQ DTCS Kit as recommended by manufacturer, or by custom sequencing service. Sequences obtained were compared with NCBI IgBLAST® and analyzed against VBASE2 Ig database [36]. Multiple sequence alignment was performed by ClustalW® from EMBL-EBI server with following default conditions: matrix, BLOSUM; gap opening penalty, 10.0; gap extension penalty, 0.05; gap separation penalty, 8; maxdiv, default; no end gap separation penalty. Alignment in the CDR3 was further adjusted manually in accordance with physical property of amino acid residues.

### PhageELISA

PhageELISA was performed as described previously [35]. Briefly, the phageELISA assay was carried out in a 96-well ELISA plate which was pre-coated with an antigen (0.3–50 µg). After incubation with 100 µl of scFv-phages at 37°C for 1 hr, in the absence or presence of free ligand, bound phages were detected by incubation with 100 µl of a horseradish peroxidase-conjugated anti-M13 mouse antibody (GE Healthcare) at 37°C for 1 hr. Activity of horseradish peroxidase was measured by a colorimetric method with *o-*phenylenediamine/H_2_O_2_ as substrates. Color was allowed to develop for 1 hr at room temperature, and absorbance at 450 nm was measured with a µQuant™ micro-plate reader (Bio-Tek).

### His-tagged scFv expression

For expression analysis, scFv clones were subcloned into pCantab His vector, transformed into *E Coli* HB2151, and periplasmic His-tagged scFvs were purified using Ni-NTA agarose beads as described in our previous publication [35].

### Recombinant N protein

Recombinant SCoV-N protein was cloned, bacterially expressed and purified as described in our previous publication [34].

## Results

### Retrieval of variable regions of germline rearranged Ig genes

**Figure 1:**
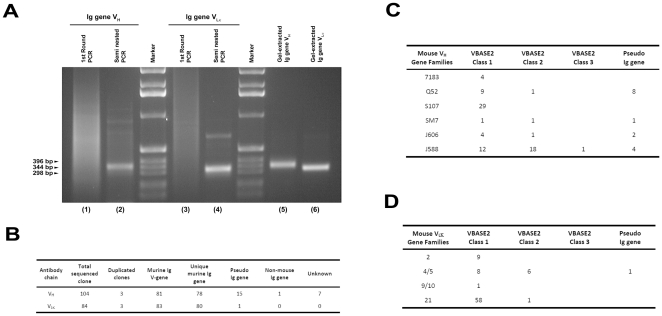
Retrieval of the variable region of rearranged germline immunoglobulin genes by semi-nested PCR. (A) Genomic DNA (200 ng) from CD19^+^ splenocytes was used as templates for semi-nested PCR to retrieve the variable region of rearranged Ig genes. The 1^st^ round PCR products of heavy- (lane 1) and κ light- (lane 3) chains were used as templates for the 2^nd^ round semi-nest PCR (lane 2 and 4). Sizes of PCR products were estimated against the 1 Kbp DNA ladders (Marker). Primers used and PCR protocols are described in Methods. After gel purification, the 2^nd^ round semi-nested PCR products of heavy- (lane 5) and κ light- (lane 6) chains were cloned into Topo TA or pGEM-T vectors for nucleotide sequence determination. (B) Sequence analysis indicates that 92% and 100% of the randomly picked V_H_ or V_Lκ_ clones are mouse immunoglobulin genes, respectively. The sequence V_H_ (C) or V_Lκ_ (D) clones were further analyzed against the VBASE2 Ig database, indicating all the sequenced clones are germline-derived. Class 1 genes are with genomic and rearranged evidence; Class 2 genes are with genomic evidence only; Class 3 genes are with rearranged evidence only as defined by VBASE2.

To retrieve the variable regions of rearranged Ig genes from genomic DNA, degenerate primer pair that is complementary to the antibody framework segment 1 (FR1) and FR4 of immunoglobulin chain was used for semi-nested PCR. As shown in Figure 1A, semi-nested PCR generated DNA fragments that displayed a size corresponding to V_H_ (lanes 2 and 5) and V_Lκ_ (lanes 4 and 6) variable segments. To confirm the DNA fragments being Ig genes, the PCR products deriving from splenocytic genomic DNA of naïve non-immunized mice were gel-purified and then cloned into vectors. The V_H_ or V_Lκ_ transformants (∼100 each) were randomly picked for sequencing. Based on successfully sequenced clones, 92.0% (96/104) and 100% (84/84) of clones were found to be murine Ig V_H_, and murine Ig V_Lκ_, respectively (Figure 1B).

To analyze the diversity of retrieved Ig genes and to confirm their germline origin, V_H_ and V_Lκ_ clones were searched for homology with germline Ig variable region genes against the VBASE2 database (Table S1). Sequence analysis indicated that the retrieved V_H_ and V_Lκ_ genes were mainly derived from 6 out of the 15 V_H_ (Figure 1C) and 4 out of the 19 V_Lκ_ (Figure 1D) subfamilies, respectively. Intriguingly, in some case the number of Ig clone with unique sequence exceeded the number of germline variable region genes. For instance, a total of 59 unique V_Lκ_ clones were identified as members of the V_Lκ_ 21 subfamily while the estimated germline Ig gene number of the V_Lκ_ 21 subfamily is only 6-13, suggesting retrieved Ig genes might have derived from both rearranged germline and hypermutated Ig genes.

### Diversification of CDR3 by frame-shifting PCR

**Figure 2:**
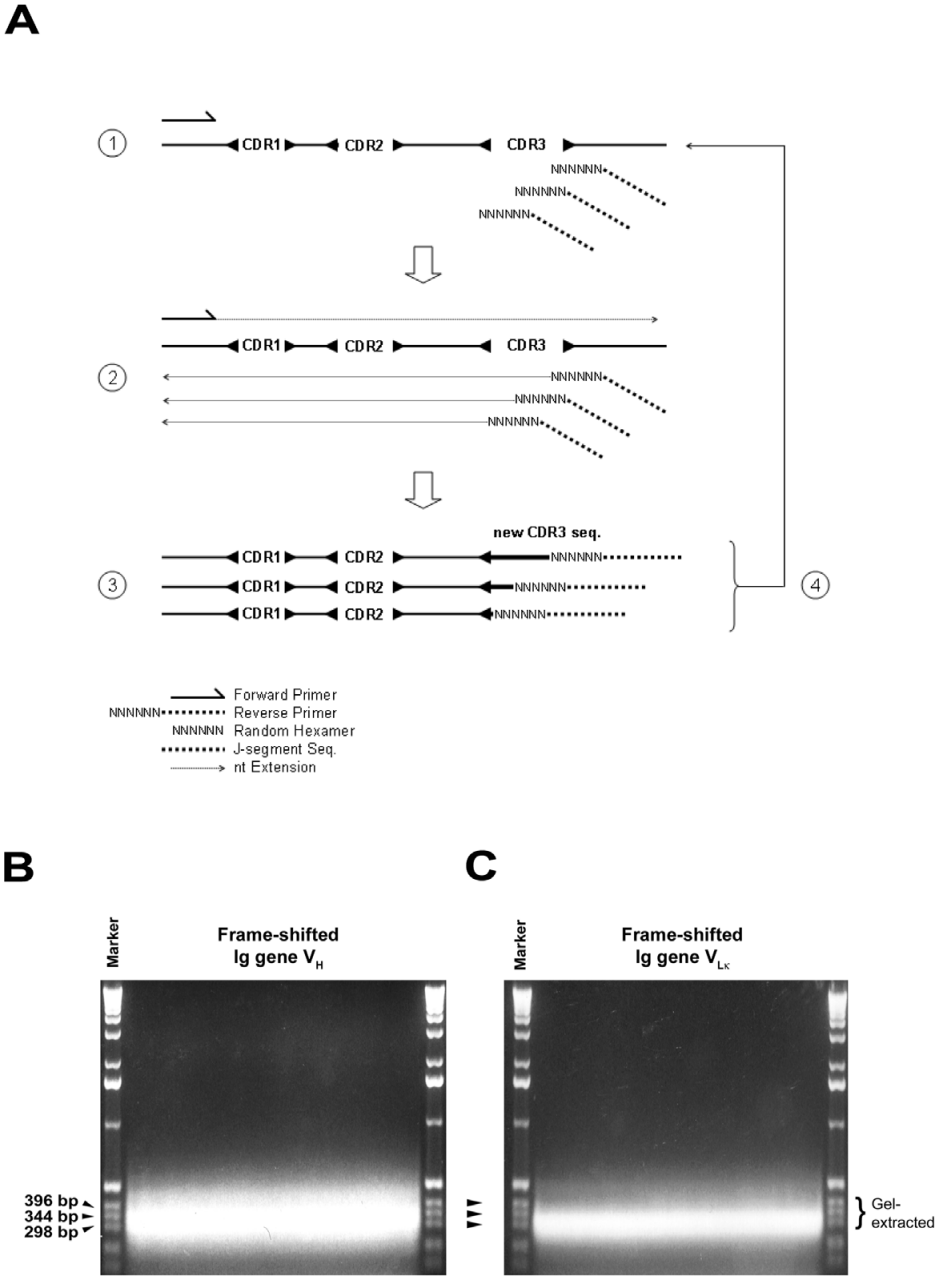
Frame-shifting PCR. (A) Schematic illustration of various steps involved in fsPCR. Imprecise pairing between the Ig FR4 framework and the degenerate J sequence of frame-shifting primer predicted the oligos annealing to multiple sites located immediately next to CDR3 region (step 1). The random hexamer portion of the frame-shifting primer allowed base pairing in CDR3 and subsequent nucleotide extension (step 2), generating library of Ig variable region fragments that are different in length and nucleotide sequence within the CDR3 (steps 3). After semi-nested PCR, 60–100 ng of purified V_H_ or V_Lκ_ (Figure 1A, lanes 5 and 6) was then subjected to fsPCR to diversify the CDR3 of heavy- (B) and κ light- (C) chains as describe in Methods. Frame-shifted heavy- and κ light-chains of size 298-396 bp were gel-extracted and used for scFv library construction.

To rescue the non-functional rearranged Ig genes and to enhance the diversity of CDR3, retrieved variable regions of Ig genes were subjected to frame-shifting PCR (fsPCR) that aimed at introducing sequences variation in CDR3. Essentially, the frame-shifting reverse primers (RFSH for V_H_ and RFSK for V_Lκ_) are composed of two portions. In the 5′-portion of the frame-shifting reverse primer is a 5′-degenerate sequence of J gene-segments while the 3′-portion is a random hexamer. We reasoned that the 5′-degenerate J gene sequence would bring the frame-shifting reverse primer close to the CDR3-J region of the Ig templates while the 3′-random hexamer would imperfectly anneal to the CDR3 (Figure 2A). As for the low sequence similarity and the sequence heterogeneity of 3′-random hexamer, annealing between templates and the frame-shifting reverses primer would not be precise and resulted in generating a library of immunoglobulin chains with different CDR3 nucleotide sequences. Indeed, as illustrated in Figure 2B, fsPCR generated DNA fragments that spread around 220-396 bp for V_H_ and 250-344 bp for V_Lκ_ Ig genes.

**Figure 3:**
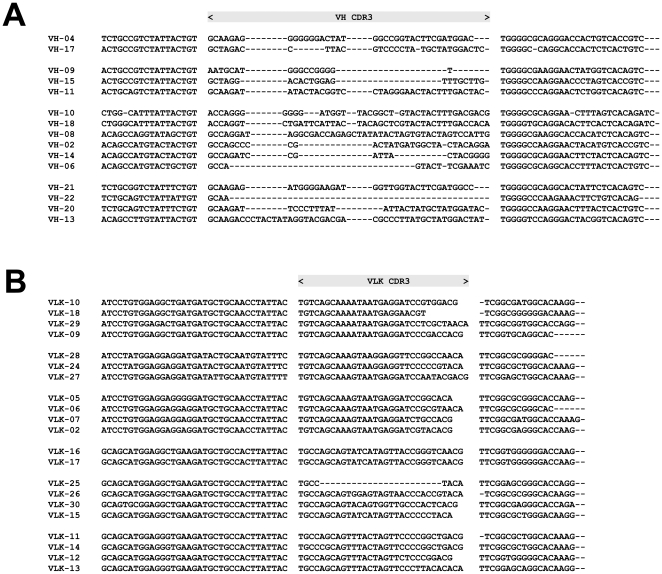
Multiple alignments of frame-shifted germline-derived V_H_ and V_Lκ_ genes. After fsPCR, the PCR products were cloned into TOPO TA cloning vector. Following nucleotide sequence determination, clones were aligned using ClustalW®. Significant sequence variations of heavy (A) and κ light (B) chains were noted in the CDR3.

To analyze the nucleotide diversity in CDR3, PCR products of fsPCR were gel-purified, subcloned and sequenced. Multiple sequence alignment of the derived V_H_ and V_Lκ_ sequences indicated a significant sequence variation within the CDR3 among various clones. It is of interest to note that sequence variation occurred mainly at the 3′-end of CDR3 which the random hexamer portion of the degenerate reverse primer was predicted to anneal to during fsPCR (Figure 3).

### Library construction and selection of phOx-selective scFvs

To evaluate the feasibility of using germline rearranged Ig genes for making a target-specific antibody, an immunized germline antibody library was constructed and then characterized against the hapten antigen 4-ethoxymethylene-2-phenyl-2-oxazolin-5-one (phOx). Three days after the third boost injection of mixture consisting of phOx-chicken serum albumin (CSA) conjugate-and incomplete Freund's adjuvant, splenocytic CD19^+^ B cells (∼0.8×10^6^) were isolated and used for retrieving variable regions of germline rearranged Ig genes by semi-nested PCR. Following CDR3 diversification by fsPCR, scFvs were assembled by overlap extension PCR and later cloned into pCANTAB 5E phagemid to establish a small scFv phage display library (5.16×10^5^ recombinants).

**Figure 4:**
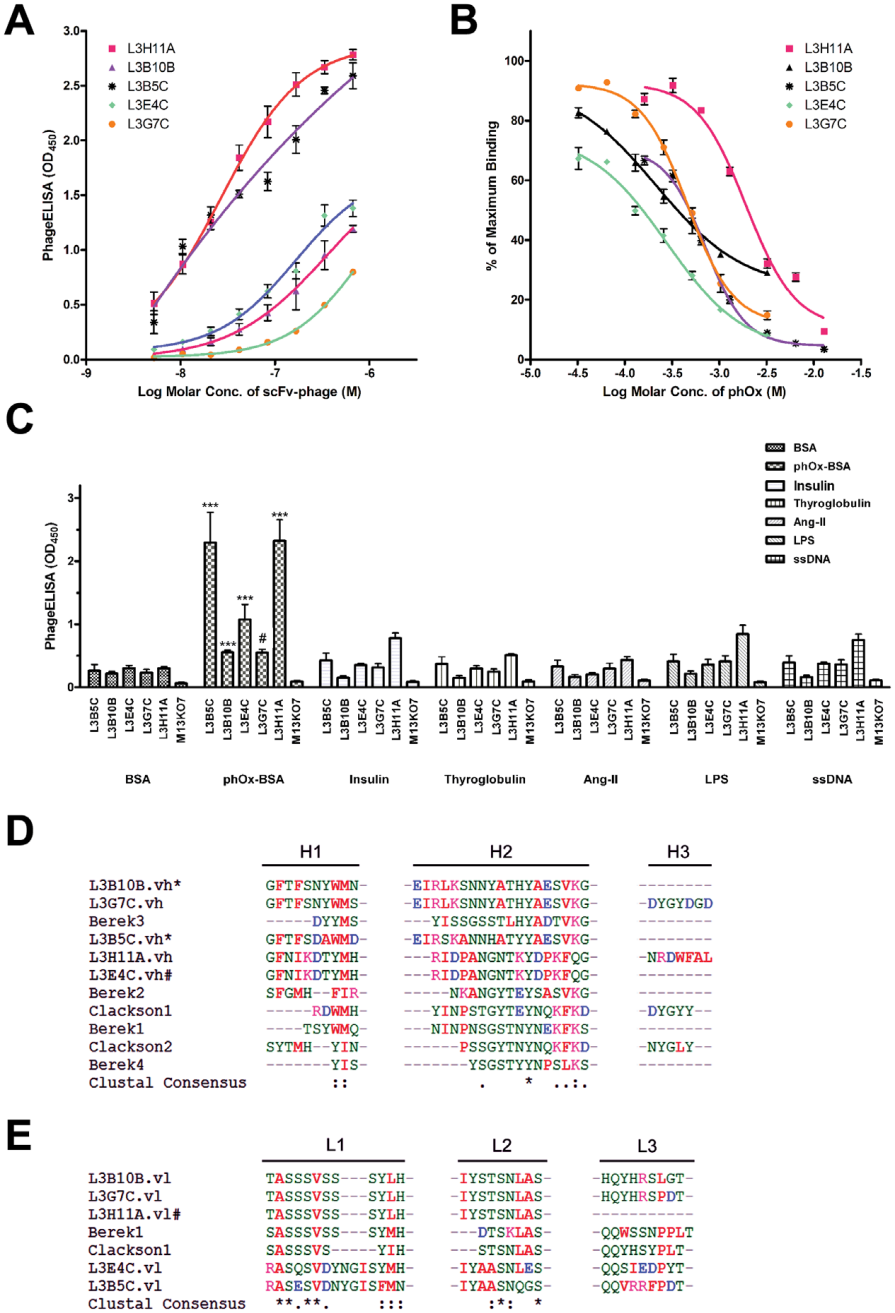
Characterization of anti-phOx scFv-phages selected from immunized germline phage-displayed scFv library. ELISA plate (96-well) was pre-coated with 50 µl of phOx-BSA (1 µg/µl) overnight. Freshly prepared phages were PEG-precipitated and then resuspended in Tris-buffered saline (TBS). Resuspended scFv-phage (50 µl) L3H11A (close square), L3A6C (close upward triangle), L3D11C (close downward triangle), L3B10B (close diamond), L3E2C (close circle), L3B5C (open square), L3E6C (open upward triangle), L3E4C (open downward triangle), and L3G7C (open circle) were incubated with immobilized phOx-BSA conjugates at 37°C for 1hr. (A) Dose-dependence saturation curve of selected scFv clones. Phages were resuspended at the indicated concentrations. Phage concentrations were estimated spectroscopically as described by Barbas III [55]. Error bars represent SEM of 3-6 separate experiments each performed in duplicate. (B) Competitive phageELISA analysis of phOx-selective scFv-phages. Phages were resuspended at a fix concentration (∼2.8×10^14^ cfu/ml) and incubated with immobilized phOx-BSA in the absence or presence of different molar concentrations of free phOx (32 µM to 13 mM). Results were normalized in percentage of saturation. Error bars represent SEM of three separate experiments each performed in duplicate. (C) Cross-reactivity of selected scFv clones was examined using phageELISA against different antigens, including BSA, phOx-BSA conjugate, insulin, thyroglobulin, Ang II-BSA conjugate, LPS, ssDNA as indicated. Error bars represent SEM of three separate experiments each performed in duplicate. Differences between means of binding to phOx-BSA *versus* to other unrelated antigens are evaluated by One Way ANOVA using GraphPad Prism 4. Statistical significance is indicated as *** (*P*<0.001), while # indicated no statistical significance. (D) The putative CDR amino acid sequences of derived phOx-selective scFvs were aligned with the published CDR sequences of anti-phOx antibodies. The physical properties of amino acids are color-coded as: aromatic and nonpolar A, V, L, I, P, F, W, M (red); acidic D, E (blue); basic K, R, H (black); polar G, S, T, C, Y, N, Q (green). For amino acid alignment, (*) denotes identical residues, (:) residues with conserved substitution, and (.) predominated by residues with similar physical property in the columns. For selected scFv clones, (*) indicated derived-Ig variable region without FR4 amino acid sequence, (#) Ig variable region with frame-shift within CDR3. Published anti-phOx sequences were extracted from Berek *et.al.* (37) and Clackson *et.al.* (38).

The phage-displayed scFv library was panned against immobilized phOx-BSA conjugate of which amount was reduced ten-fold in each successive round of panning. After 5 rounds of panning, 288 clones were randomly picked. Specificity of candidate scFv-phages was assessed using phageELISA against phOx-BSA conjugate, and 15 phOx-BSA binders were identified from which 5 high binding clones were selected for further characterization including L3B8C, L3B10B, L3E4C, L3G7C and L3H11A (Table S2). The phOx-selective phage clones bound phOx-BSA in a concentration-dependent manner (Figure 4A). The binding of these phage clones to phOx heptan was further confirmed by competitive phageELISA in which free phOx competed with immobilized phOx-BSA for phage binding. Phage binding towards immobilized phOx-BSA was dose-dependently suppressed by free phOx at concentrations that spread more than two log_10_ scales, suggesting each phage clone exhibited distinct binding characteristics towards phOx (Figure 4B). Furthermore, the phage clones bound significantly higher to phOx-BSA than to other irrelevant immobilized antigens including BSA, insulin, thyroglobulin, angiotensin II (Ang II), LPS and ssDNA (Figure 4C), suggesting the isolated phage clones were selective towards phOx. To compare the phOx binding pocket, putative amino acid sequences of the phOx-selective scFvs were translated, and their CDRs were identified and aligned. It is noted that some of the identified scFvs shared conserved CDR motifs with previously characterized phOx-binders in the Ig database [37], [38], while others showed conservative amino acid substitutions (Figure 4, panels D and E, Table S2).

### ScFv library derived from a single scFv template

**Figure 5:**
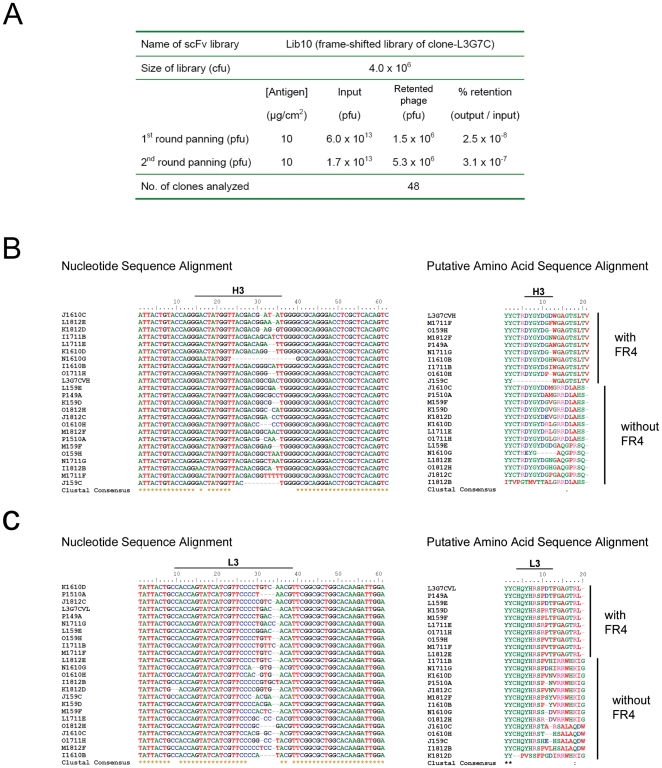
Panning performance and nucleotide sequence analysis of single temple-derived scFv library. Phage clone L3G7C with moderate affinity towards phOx was subjected to fsPCR and the products were used to construct a phage-displayed scFv library. (A) Panning performance of L3G7C-derived scFv library against immobilized phOx-CSA conjugate. Phage clones of the L3G7C-derived scFv library were randomly picked for sequence determination. Nucleotide and putative amino acid sequences of the (B) heavy- and (C) κ light-chain CDR3 (H3 and L3, respectively) were aligned. Nucleotide sequence variation was noted in the CDR3, and lack of consensus antibody FR4 was noted in some of the phage clones.

It was reasoned that if fsPCR could truly diversify CDR3, an scFv library could be established from a single scFv template. To test this hypothesis, a new scFv library was constructed using a single scFv clone L3G7C that exhibited a moderate phOx-specific binding and a noticeable non-specific binding towards LPS (Figure 4). After V_H_ and V_Lκ_ amplification and CDR3 diversity enhancement by fsPCR, the newly constructed scFv library was panned against phOx-BSA conjugate again. Two rounds of panning were carried out and 48 candidate clones were randomly picked (Figure 5A). The nucleotide sequence of selected phage clones were determined, and sequence alignment indicated that sequence variations were found only within the CDR3 of both V_H_ (Figure 5B) and V_Lκ_ (Figure 5C). Furthermore, it was noted that some of the selected phOx-binders exhibited frame-shift in the Ig CDR3 coding sequences, and resulting in lacking of consensus CDR3 boundary sequence and without recognizable framework 4 (FR4) of Ig (Figure 5 and Table S3).

**Figure 6:**
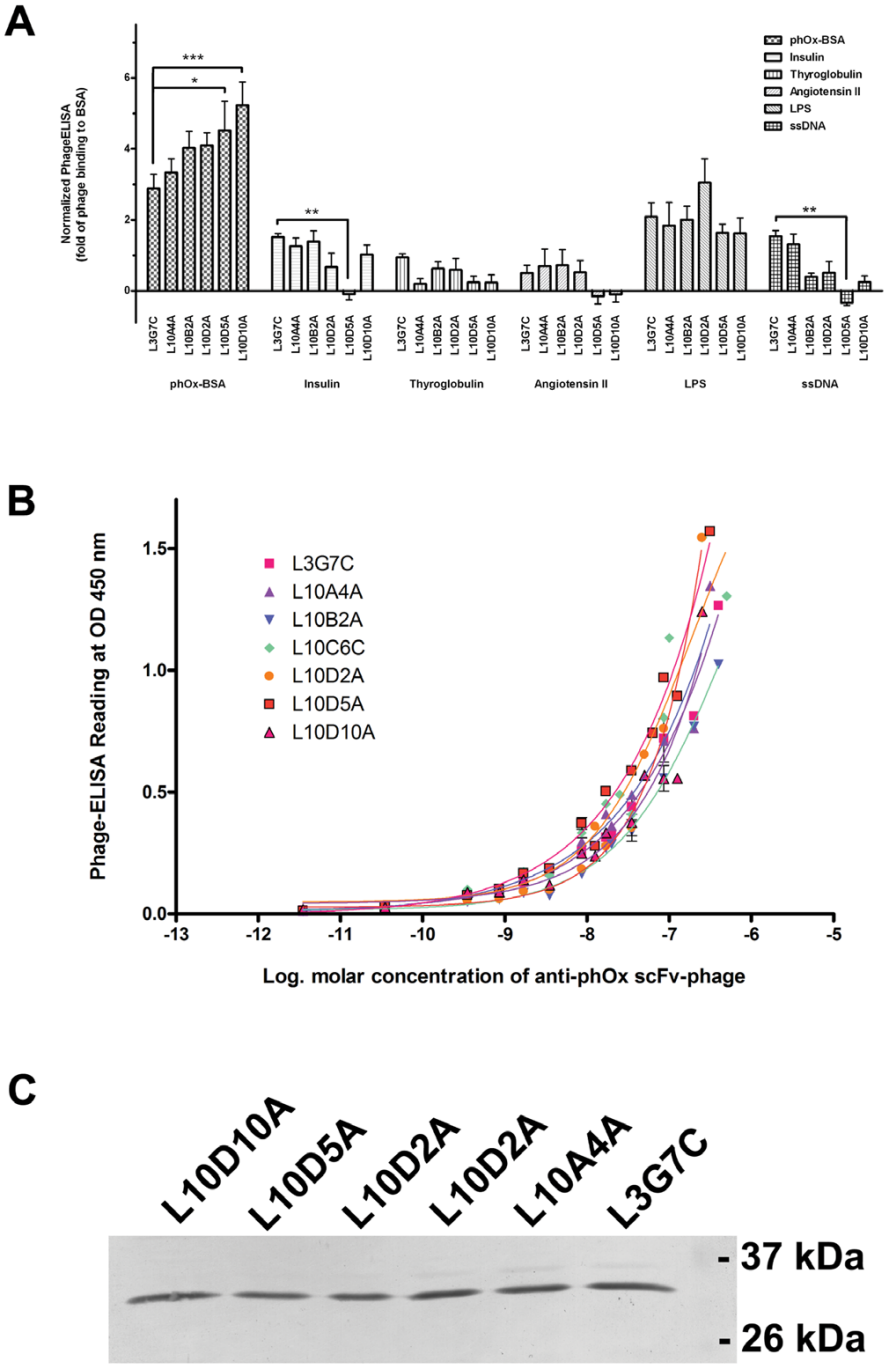
Characterization of selected scFv clones obtained from L3G7C-derived scFv library. (A) The specificity of anti-phOx scFv clones that derived from L3G7C was evaluated using phageELISA against different antigens, including phOx-BSA conjugate, insulin, thyroglobulin, Ang II-BSA conjugate, LPS, ssDNA as indicated. After subtraction of the background binding of M13KO7 helper phage, the binding activity of scFv clones towards different antigens was normalized against their binding towards BSA (negative control). Error bars represent SEM of three separate experiments each performed in duplicate. Difference of the means between selected clones and the parental clone (L3G7c) is evaluated by One Way ANOVA using GraphPad Prism 4. Statistical significance is indicated as *** (*P*<0.001); ** (*P*<0.01) and * (*P*<0.05). (B) PhageELISA analysis of phOx-selective scFv-phages. ELISA plate (96-well) was pre-coated with 50 µl of phOx-BSA (1 µg/µl) overnight. Resuspended scFv-phage clones L3H11A (close square), L3A6C (close upward triangle), L3D11C (close downward triangle), L3B10B (close diamond), L3E2C (close circle), L3B5C (open square), L3E6C (open upward triangle), L3E4C (open downward triangle), and L3G7C (open circle) at the indicated concentration were added and incubated with immobilized phOx-BSA at 37°C for 1hr. Error bars represent SEM of 3-6 separate experiments each performed in duplicate. (C) Phage clones were subcloned into a pCantab His vector for adding a His_6_ tag at the C-terminus of scFv and bacterial periplasmic expression. After Ni-NTA purification, expressions of scFv proteins was examined by Western protein analysis, and the protein blot was probed with an anti-His antibody.

Reactivity of the selected clones was further evaluated by phageELISA, and 7 distinct clones that exhibited high phageELISA signal were identified (Table S4). PhageELISA indicated the selected clones bound phOx avidly with minimum binding to other control antigens, except LPS (Figure 6A). There was no apparent changes in specific binding (Figure 6B), but clones L10D5A and L10D10A gave a significant lower binding than the parental L3G7C to the unrelated antigens insulin, angiotensin and ssDNA (Figure 6A). Despite clones L10D5A and L10D10A still gave noticeable non-specific binding towards LPS, the binding was substantially lower than the parental L3G7C. These results suggested that fsPCR diversified the CDR3 sequences, which resulted in decreasing the non-specific binding and therefore enhancing the signal-to-noise ratio of antigen binding. Hence, fsPCR could be used for optimizing the binding selectivity of antibody.

Consistent with previous reports that unexpected frame-shifts and stop codons are commonly found in gene expressed in phage-display system [39], [40], sequence analysis indicated that some of the selected phage clones contained stop codon or displayed a frame-shift in the coding region (Table S4). To confirm the expression of scFv proteins, those selected phage clones were subcloned into a pCantab His vector which also fused a His_6_ tag to the C-terminus of scFv protein. The clones were bacterially expressed and the periplasmic proteins were collected. Following Western protein analysis, His_6_-tagged recombinant scFv proteins with a predicted molecular size of ∼30 kDa were detected (Figure 6C), suggesting the L3G7C-derived phOx-selective phage clones were expressed properly in spite of the coding region containing stop codon and/or frame-shift.

### Antigen-specific antibody fragment derived from naïve verse immunized splenocytic germline scFv library

To determine whether germline antibody library represented the immune status, a naïve phage-displayed scFv library (L4; 3.5×10^5^ recombinants) was constructed using splenocytic DNA collected from non-immunized mice, and the library was screened against recombinant N protein of SARS-associated coronavirus (SCoV). For comparison, an immunized germline scFv library (L9; 3×10^6^ recombinants) was also prepared using splenocytic genomic DNA from mice immunized with heat-inactivated SARS associated coronavirus. After two rounds of biopanning, 2100 and 96 specific anti-N scFvs were isolated from both the immunized and naïve libraries, respectively. Following confirmation with phageELISA, six strong binders for SCoV-N protein were identified from naive (L4A3A; L4A3B, L4A8B) and immunized (L9A6B, L9A11B, and L9N01) libraries. It is of interest to note that not only a larger number of phage clones was isolated, 9 clones with identical sequence were found from immunized library L9 and those clones were subsequently referred as L9N01.

**Figure 7:**
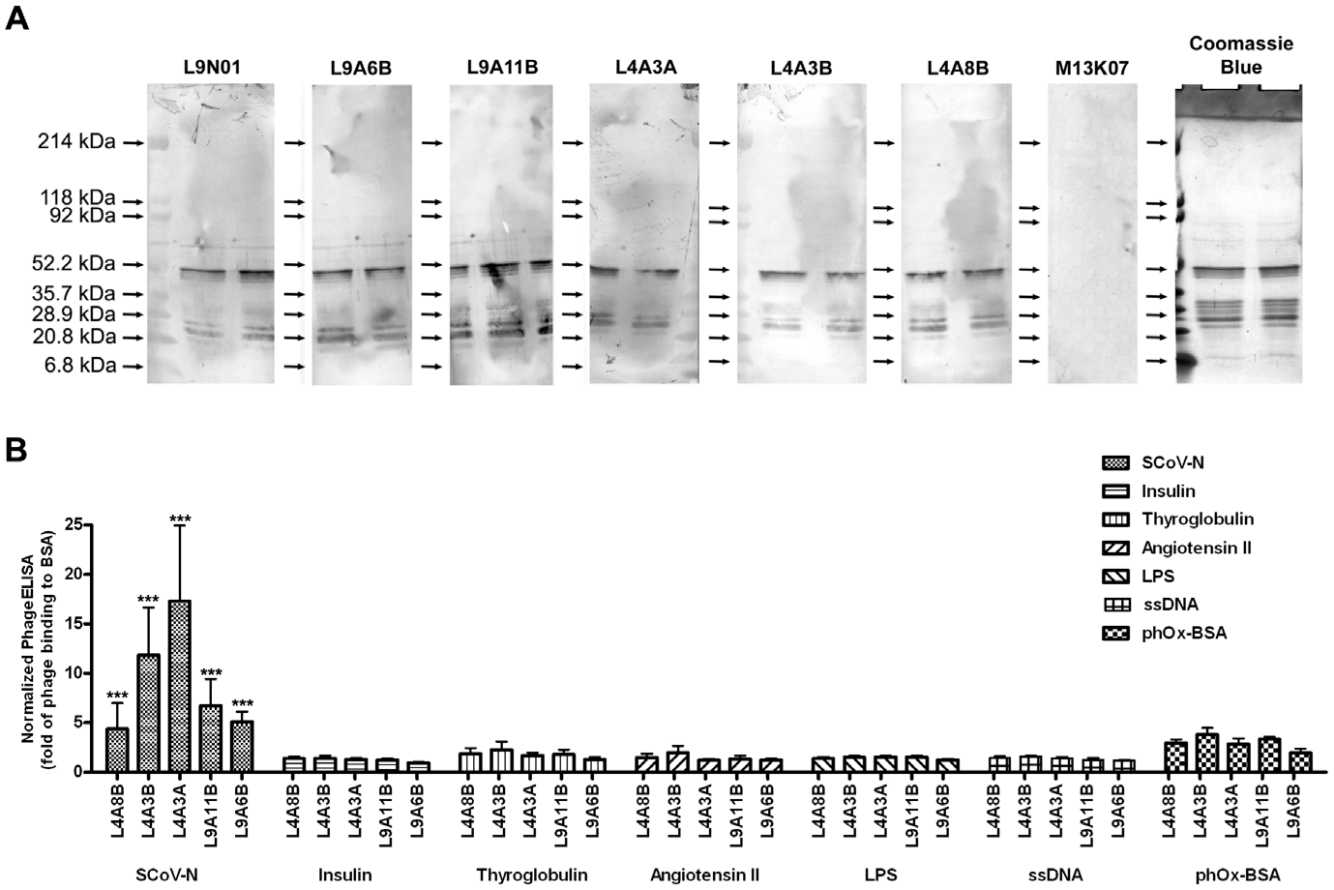
Characterization of anti-N scFv-phages. (A) Western protein analysis of scFv-phage binding to recombinant SCoV-N protein. Purified His_6_-SCoV-N recombinant protein was separated on gradient sodium dodecyl sulfate (5% - 20%) polyacrylamide gel and transferred onto a 0.2 µm nitrocellulose membrane. The stripes were probed with different anti-N scFv-phages, including L9A6B, L9A11B, L4A3A, L4A3B, L4A8B, and M13KO7 helper phage (negative control) as indicated. Purified SCoV-N protein was stained with Coomassie Blue for reference. (B) Cross-reactivity of selected anti-N scFv clones was evaluated using phageELISA against different antigens, including BSA, SCoV-N, insulin, thyroglobulin, Ang II-BSA conjugate, LPS, ssDNA as indicated. Error bars represent SEM of three separate experiments each performed in duplicate. Difference between means of binding to SCoV-N protein *versus* to other unrelated antigens is evaluated by One Way ANOVA using GraphPad Prism 4. Statistical significance is indicated as *** (*P*<0.001).

**Figure 8:**
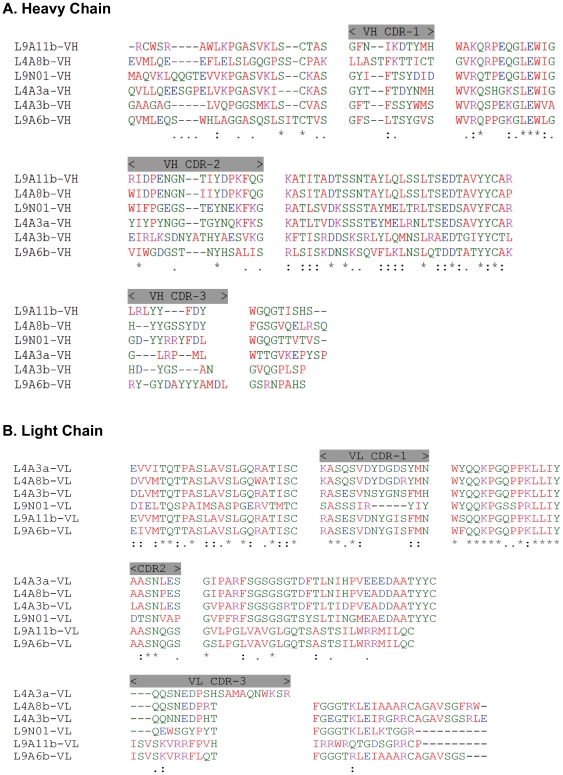
Alignment of anti-N scFv clones. Putative amino sequences of selected anti-N scFvs were aligned using ClustalW®, and the CDRs were indicated. Physical properties of amino acids are color-coded indicating: aromatic and nonpolar A, V, L, I, P, F, W, M (red); acidic D, E (blue); basic K, R, H (black); polar G, S, T, C, Y, N, Q (green). Symbols under the alignment indicate: identical residues (*); conserved (.) and semi-conserved (:) substitutions.

In addition to recognizing recombinant SCoV-N protein in its native form, the selected anti-N scFv-phages also bound to the recombinant N protein in Western blot (Figure 7A). Binding of the selected scFv-phages was specific as it only exhibited high binding towards recombinant N protein but not to any of the non-specific controls (Figure 7B). However, it was noted that the phage clone L9N01 cross-reacted with interleukin 11 [34]. Sequence analysis indicated that those selected anti-N scFvs were derived from different rearranged Ig variable region genes, and higher sequence homology was found amongst the light chains (Figure 8, Table S5). However, there was on distinct difference between clones derived from naïve or immunized libraries, except more positive clones were isolated from the immunized library.

## Discussion

In this proof-of-concept study, we have demonstrated the possibility of using rearranged germline immunoglobulin variable region genes as the source of antibody repertoires for construction of germline scFv library. In order to retrieve the rearranged variable region genes from genomic DNA, degenerate primers were used. Though changes in the nucleotide sequences of FR1 and FR4 is a major disadvantage of using degenerate primers, sequence-alteration by degenerate primers was considered as a minor influence [41]. On the other hand, the use of family-specific primers assures less change in nucleotide sequences, but a large number of family specific primer pairs will be required for retrieving the Ig variable region genes from genomic DNA. Moreover cross-family amplification cannot be avoided if all the primers are used at the same time.

The incorporation of a random hexamer in the fsPCR reverse primer may cause a bias towards GC-rich sequences. Nucleotide sequence analysis revealed that the AT and GC contents in the V_H_ CDR3 were 53 and 47% (data not shown), respectively, suggesting that there was no significant bias towards GC-rich sequences. Furthermore, the bias of PCR condition that favored certain nucleotide sequences was minimized by adding 5% DMSO. Although an addition of both DMSO (5%) and betaine (1 M) was suggested to achieve a uniform amplification of heterogeneous templates [42], we used 5% DMSO alone in the present study because the yield of PCR amplification from genomic templates was not satisfactory in the presence of both chemicals.

Sequence analysis indicated that the variable regions of germline Ig genes were successfully retrieved from genomic DNA. Furthermore, retrieved Ig variable regions could be grouped into 4 V_Lκ_ and 7 V_H_ subgroups with reference to germline V gene-segment (Table S6). It is of interest to note that more V_H_ (7 out 15) subfamilies were retrieved than that of V_Lk_ (4 out of 19) using present strategy. However, when comparing the distributions of Ig clones in each V genes subfamily, distributions of Ig clones of the naive germline library were different from that of the target-specific Ig clones (Table S6). These results are consistent with the notion that Ig repertoires of naive germline scFv library are highly diverse while library derived from immunized mice was an image of humoral response to antigens.

*In silico* translation of the coding sequences indicated that some selected scFvs contained unexpected frame-shifts and stop codons. However, protein expression study showed that those clones with defective ORF produced normal scFv recombinant protein in *E. Coli*. Previously, it has been reported that unexpected frame-shifts and stop codons were found in genes expressed in the phage-displayed system [39], [40]. A eukaryotic yeast-displayed system has been used to identify antigen-specific scFv clones from a human non-immune antibody library, overcoming the various shortcomings of phage-displayed system [43]. Hence, to facilitate the cloning and production of antigen-specific scFvs, the germline antibody library could alternatively be expressed on the yeast-displayed system.

In agreement with previous report that the length of mice heavy chain CDR3 ranges from 2-19 residues with an average size of 10 residues [44], the heavy chain CDR3 length of those germline-derived anti-phOx and anti-N scFvs ranged from 2 to 23 residues with a predominant distributions at 8 - 11 residues (Table S7). Consistent with light chain lacking the D segment of heavy chain, the light chain CDR3 length was smaller than that of heavy chain and ranged from 8 to 18 residues with a peak value at 9 residues (Table S7). The similarity in CDR3 length distribution between native and recombinant antibodies suggested the hypervariable regions that derived from germline rearranged Ig genes were folded into the canonical structure of immunoglobulins [27].

Multiple sequence alignment of those isolated anti-phOx scFv clones indicated that the nucleotide sequences in CDR1 and CDR2 of both heavy and light chains were highly conservative, and indeed they shared significant homology with previously characterized anti-phOx antibodies. This result is consistent with previous reports that mouse anti-phOx IgG response is highly restricted and most antibodies are preferentially generated via the recombination of V_H_-Ox1 and V*_κ_*-Ox1 variable gene segments [23], [37]. In comparison with the CDR3 of heavy chain, the light chain CDR3 was more conservative. This result suggests that light chain CDR3 of anti-phOx antibody may play a critical role in phOx binding. The less variability in light chain CDR3 is also in line with previous observation that in the course of affinity maturation of anti-phOx antibodies, hypermutations of amino acid residues in light chain are much concentrated in the CDR1 rather than the CDR3 [45]. It has been reported that the CDR3 of anti-phOx heavy chain consists of a sequence motif Asp-X-Gly-X-X (where X is any amino acid) [38], [46] and the length of CDR3 ranges from 4-11 amino acid residues [47]. In the present study, it is noted that majority of the heavy chain CDR3 of anti-phOx scFvs were 8-11 amino acid residues in length (Tables S2 and S6). The heavy chain CDR3 of L3G7C consisted of the sequence motif Asp-X-Gly-X-X, but other clones were not. These results suggest that the anti-phOx scFvs prepared from genomic DNA with fsPCR displayed similar characteristics of anti-phOx antibody derived from hybridoma or mRNA. However, by comparing the CDR3 composition, anti-phOx scFvs derived from genomic DNA with fsPCR were more heterogeneous in nature.

Moreover, with fsPCR mimicking somatic recombination, we successfully generated CDR3 heterogeneity in length and nucleotide sequence among those anti-phOx scFvs that were obtained from the L3G7C-derived scFv library (Table S4). In comparison with the parental L3G7C, those single template-derived anti-phOx scFvs gave a lower non-specific binding, suggesting fsPCR can be used in antibody engineering for optimizing antibody selectivity.

Sequence variation was found mainly localized at the 3′-end of CDR3 among the anti-phOx scFvs which exhibited different non-specific binding, suggesting the amino acids in the C-terminal end of CDR3 played a critical role in determining the reactivity of antibody. Indeed, the result is consistent with the observation that the last three amino acids of CDR3 in llama antibody determining the reactivity towards different subtype of HIV strain [48]. However, the molecular nature of how these terminal amino acids modulate the binding between antibody and antigen remains to be examined.

Consistent with the results of anti-phOx scFvs, light chain of the anti-N phage clones shared higher homology. On the other hand, higher diversity of amino acids in the CDR3 region of heavy chain was noted. In spite of lacking noticeable difference among the anti-N phage clones that obtained from the naïve and immunized libraries, clones with identical sequence were enriched in the immunized library. These results suggest that antigen-specific monoclonal antibody could be easily obtained from the immunized germline library bypassing the labor-intensive hybridomas preparation.

In principle, the germline scFv library is also an effective means for profiling an antibody response by retrieving rearranged immunoglobulin genes from the CD19^+^ splenocytes that are stimulated by antigen and undergo clonal expansion, which provides a much-in-demand tool for analyzing the humoral responses to antigens. Indeed, using a germline scFv library derived from splenocytic DNA of mice immunized with heat-inactivated SARS-coronavirus, we have identified an anti-N scFv that cross-reacts with interleukin 11 [34].

Comparing with the conventional mRNA-derived antibody libraries, germline rearranged Ig gene-derived library may consist of antibodies that have not undergone hypermutations, which results in generating polyreactive antibodies [49]. Indeed, despite phOx-selective binders were successfully isolated from the germline antibody libraries, some selected anti-phOx scFvs exhibited noticeable binding to unrelated antigens. On the other hand, the use of genomic DNA will benefit from its higher stability in handling and larger feasibility in sampling. In addition, the V gene repertoires in the germline antibody library are predicted to be wider than that of mRNA-derived library. For instance, heavy and light chain clones deriving from V pseudogenes (Table S1) were found in the present study. Furthermore, among those isolated anti-phOx scFvs, we found CDR sequences that had been reported previously, and we also identified new CDR motifs. However, in order to evaluate the repertoire diversity of the germline scFv library and to unveil the molecular nature that determines antibody binding specificity, the germline antibody library should be panned against a panel of antigens, and selected antibodies could then be compared for their binding specificity and binding characteristics.

Insertions and deletions of CDR3 nucleotides, which expand the repertoires of antibody hypervariable loop both in length and in structure, have recently been shown to be an additional mechanism how immunoglobulin V region genes are diversified [50], [51], [52]. Moreover, lines of evidence suggest that diversity of CDR3 plays a critical role in antigen recognition [29], [30]. These results support the notion that diversity enhancement of CDR3 by fsPCR can expand antibody diversity beyond what is encoded by germline Ig genes [21]. Current strategy can achieve the effects of gene-shuffling, site-directed mutagenesis, and nucleotide(s) deletion/insertion in one fsPCR step, which not only rescues stop-codon containing Ig genes from the genome, but also modify the repertoires of Ig genes, simultaneously. On the other hand, previous studies have indicated that the CDR3 loop of heavy chain is potentially immunogenic [53], [54], diversity enhancement of CDR3 by fsPCR may further increase the immunogenicity of antibody.

Taken together, a simple PCR-based method has been developed to use rearranged germline Ig genes as source of antibody repertoires for library construction. This germline antibody library allows us effectively to prepare target-specific antibody and to profile antibody response.

## Supporting Information

Figure S1 **Schematic diagram showing primer locations.** In reference to an scFv molecule, locations of primers used in present study are indicated together with their nucleotide sequences. Restriction sites for cloning in the primers are underlined and restriction enzymes are indicated.
(TIF)

Table S1 **Analysis of immunoglobulin clones derived from mouse genomic DNA.**
(XLS)

Table S2 **Analysis of anti-phOx scFv clones from immunized mice-derived scFv Lib03.**
(XLS)

Table S3 **Putative reading framework of Library 10 clones that derived from a single scFv template L3G7C.**
(DOC)

Table S4 **Analysis of anti-phOx scFv from L3G7C-derived scFv Lib10.**
(XLS)

Table S5 **Analysis of anti-SCoVN scFv derived from scFv Lib04 (naive) and Lib09 (immunized).**
(XLS)

Table S6 **Germline V-minigene Usage.**
(XLS)

Table S7 **Number of amino acid residue in CDR3.**
(DOC)
