# Supplementary figures and images for: Construction and Characterization of Single-Chain Variable Fragment Antibody Library Derived from Germline Rearranged Immunoglobulin Variable Genes
Source: PLoS One. 2011 Nov 11;6(11):e27406. doi: 10.1371/journal.pone.0027406 (PMC3214059; doi:10.1371/journal.pone.0027406)

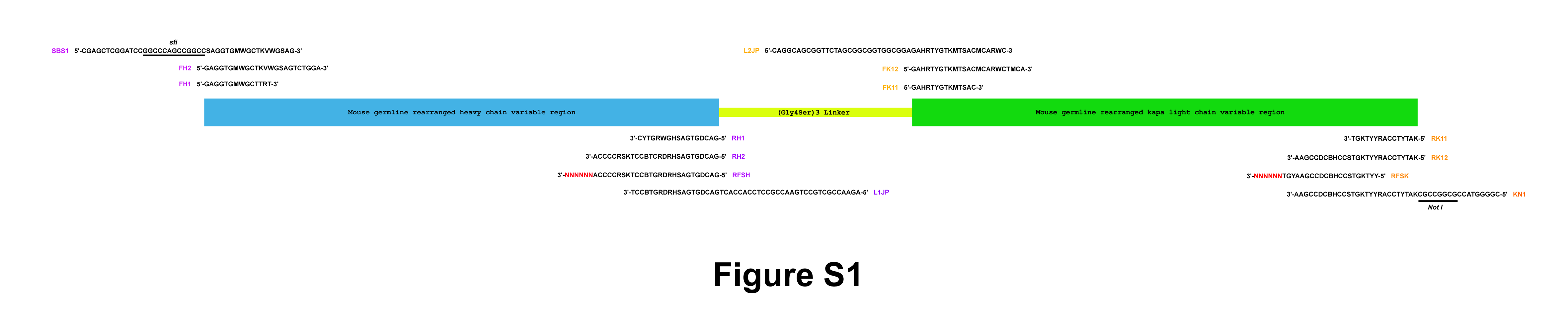

Supplement: Figure S1 — Schematic diagram showing primer locations. In reference to an scFv molecule, locations of primers used in present study are indicated together with their nucleotide sequences. Restriction sites for cloning in the primers are underlined and restriction enzymes are indicated. (TIF) [file pone.0027406.s001.tif]
