# Supplementary material for: Construction and Characterization of Single-Chain Variable Fragment Antibody Library Derived from Germline Rearranged Immunoglobulin Variable Genes
Source: PLoS One. 2011 Nov 11;6(11):e27406. doi: 10.1371/journal.pone.0027406 (PMC3214059; doi:10.1371/journal.pone.0027406)
Supplement: Table S3 — Putative reading framework of Library 10 clones that derived from a single scFv template L3G7C. (DOC) [file pone.0027406.s004.doc]

**Table S3: Putative reading framework of Library 10 clones that derived from a single scFv template L3G7C.**

***A. Heavy chain without reading framework shift***

>L3G7CVH

tattactgtaccagggactatggttacgacggcgactggggcgcagggacctcgctcaca

Y Y C T R D Y G Y D G D W G A G T S L T

>I1711B

tattactgtaccagggactatggttacgacagcatctggggcgcagggacctcgctcaca

Y Y C T R D Y G Y D **S I** W G A G T S L T

>P149A

tattactgtaccagggactatggttacgacggcgcctggggcgcagggacctcgctcaca

Y Y C T R D Y G Y D G A W G A G T S L T

>I1610B

tattactgtaccagggactatggttacgacgggcattggggcgcagggacctcgctcaca

Y Y C T R D Y G Y D G H W G A G T S L T

>M1711F

tattactgtaccagggactatggttacgacggtttttggggcgcagggacctcgctcaca

Y Y C T R D Y G Y D G F W G A G T S L T

>N1711G

tattactgtaccagggactatggttacgacggctaatggggcgcagggacctcgctcaca

Y Y C T R D Y G Y D G - W G A G T S L T

>M1812F

tattactgtaccagggactatggttacgacggcaactggggcgcagggacctcgctcaca

Y Y C T R D Y G Y D G N W G A G T S L T

>O1610H

tattactgtaccagggactatggttacgacccc---tggggcgcagggacctcgctcaca

Y Y C T R D Y G Y D P - W G A G T S L T

>J159C

tattac------------------------------tggggcgcagggacctcgctcaca

Y Y - - - - - - - - - - W G A G T S L T

(8/21 sequenced heavy chain clones)

***B. Heavy chain with putative reading framework shift***

>L3G7CVH

tattactgtaccagggactatggttacgacggcgactggggcgcagggacctcgctcaca

Y Y C T R D Y G Y D G D W G A G T S L T

>N1610G

tattactgtaccagggaatatggtgcgcagggacctcgctcacag

Y Y C T R E Y G A Q G P R S Q

>M159F

tattactgtaccagggactatggttacgacggagtggggcgcagggacctcgctcacagt

Y Y C T R D Y G Y D G V G R R D L A H S

>K159D

tattactgtaccagggactatggttacgacggcgtggggcgcagggacctcgctcacagt

Y Y C T R D Y G Y D G V G R R D L A H S

>O1711H

tattactgtaccagggactatggttacgacgggttggggcgcagggacctcgctcacagt

Y Y C T R D Y G Y D G L G R R D L A H S

>J1610C

tattactgtaccagggactatggttacgacgatatggggcgcagggacctcgctcacagt

Y Y C T R D Y G Y D D M G R R D L A H S

>K1610D

tattactgtaccagggactatggttacgacaggttggggcgcagggacctcgctcacagt

Y Y C T R D Y G Y D R L G R R D L A H S

>I1812B

tattactgtaccaggaactatggttacaacggcattggggcgcagggacctcgctcacag

Y Y C T R N Y G Y N G I G A Q G P R S Q

>J1812C

tattactgtaccagggactatggttacgacggacctggggcgcagggacctcgctcacag

Y Y C T R D Y G Y D G P G A Q G P R S Q

>L1812E

tattactgtaccagggactatggttacgacggaaatggggcgcagggacctcgctcacag

Y Y C T R D Y G Y D G N G A Q G P R S Q

>K1812D

tattactgtaccagggactatggttacgacgaggtggggcgcagggacctcgctcacagt

Y Y C T R D Y G Y D E V G R R D L A H S

>L1711E

tattactgtaccagggactatggttacgacagattggggcgcagggacctcgctcacagt

Y Y C T R D Y G Y D R L G R R D L A H S

>L159E

tattactgtaccagggactatggttacgacggcgatggggcgcagggacctcgctcacag

Y Y C T R D Y G Y D G D G A Q G P R S Q

>P1510A

tattactgtaccagggactatggttacgacgcaatggggcgcagggacctcgctcacagt

Y Y C T R D Y G Y D A M G R R D L A H S

(13/21 sequenced heavy chain clones)

***C. Light chain without reading framework shift***

>L3G7CVL

tattactgccaccagtatcatcgttcccctgacacattcggcgctggcacaagattgga

Y Y C H Q Y H R S P D T F G A G T R L

>L1711E

tattactgccaccagtatcatcgttcccgccccacgttcggcgctggcacaagattgga

Y Y C H Q Y H R S R P T F G A G T R L

>O1711H

tattactgccaccagtatcatcgttcccggcctacgttcggcgctggcacaagattgga

Y Y C H Q Y H R S R P T F G A G T R L

>O159H

tattactgccaccagtatcatcgttcccctgttacattcggcgctggcacaagattgga

Y Y C H Q Y H R S P V T F G A G T R L

>L1812E

tattactgccaccagtatcatcgttcccctgtcacattcggcgctggcacaagattgga

Y Y C H Q Y H R S P V T F G A G T R L

>P149A

tattactgccaccagtatcatcgttcccctgacacattcggcgctggcacaagattgga

Y Y C H Q Y H R S P D T F G A G T R L

>L159E

tattactgccaccagtatcatcgttccccggacacattcggcgctggcacaagattgga

Y Y C H Q Y H R S P D T F G A G T R L

>M159F

tattactgccaccagtatcatcgttccccactcacattcggcgctggcacaagattgga

Y Y C H Q Y H R S P L T F G A G T R L

>K159D

tattactgccaccagtatcatcgttccccacgaacattcggcgctggcacaagattgga

Y Y C H Q Y H R S P R T F G A G T R L

(8/22 sequenced light chain clones)

***D. Light chain with putative reading framework shift***

>L3G7CVL

tattactgccaccagtatcatcgttcccctgacacattcggcgctggcacaagattgga

Y Y C H Q Y H R S P D T F G A G T R L

>O1610H

tattactgccaccagtatcatcgttccacgtgacattcggcgctggcacaagattgga

Y Y C H Q Y H R S T - H S A L A Q D W

>I1812B

tattactgccaccagtatcatcgttcccccgtgctacattcggcgctggcacaagattgg

Y Y C H Q Y H R S P V L H S A L A Q D W

>K1812D

tattactgaccagtatcatcgttccccggtgacattcggcgctggcacaagattgga

Y Y - P V S S F P G D I R R W H K I G

>K1610D

tattactgccaccagtatcatcgttcccctgtcaacgttcggcgctggcacaagattgga

Y Y C H Q Y H R S P V N V R R W H K I G

>N1610G

tattactgccaccagtatcatcgttccagtgacgttcggcgctggcacaagattgga

Y Y C H Q Y H R S S D V R R W H K I G

>P1510A

tattactgccaccagtatcatcgttcccctaacgttcggcgctggcacaagattgga

Y Y C H Q Y H R S P N V R R W H K I G

>J1812C

tattactgccaccagtatcatcgttcccccgtcaacgttcggcgctggcacaagattgga

Y Y C H Q Y H R S P V N V R R W H K I G

>O1812H

tattactgccaccagtatcatcgttcccgcgacgttcggcgctggcacaagattgga

Y Y C H Q Y H R S R D V R R W H K I G

>M1812F

tattactgccaccagtatcatcgttccccctcctacgttcggcgctggcacaagattgga

Y Y C H Q Y H R S P S Y V R R W H K I G

>J1610C

tattactgccaccagtatcatcgttccacggcacgttcggcgctggcacaagattgga

Y Y C H Q Y H R S T A R S A L A Q D W

>I1610B

tattactgccaccagtatcatcgttccccatacgttcggcgctggcacaagattgga

Y Y C H Q Y H R S P Y V R R W H K I G

>I1711B

tattactgccaccagtatcatcgttcccctgttcacattcggcgctggcacaagattgga

Y Y C H Q Y H R S P V H I R R W H K I G

>N1711G

tattactgccaccagtatcatcgttcccctgaccacattcggcgctggcacaagattgga

Y Y C H Q Y H R S P D H I R R W H K I G

>J159C

tattactgccaccagtatcatcgttcccacgaacattcggcgctggcacaagattgga

Y Y C H Q Y H R S H E H S A L A Q D W

(14/22 sequenced light chain clones)
