# Supplementary material for: Construction and Characterization of Single-Chain Variable Fragment Antibody Library Derived from Germline Rearranged Immunoglobulin Variable Genes
Source: PLoS One. 2011 Nov 11;6(11):e27406. doi: 10.1371/journal.pone.0027406 (PMC3214059; doi:10.1371/journal.pone.0027406)
Supplement: Table S7 — Number of amino acid residue in CDR3. (DOC) [file pone.0027406.s008.doc]

**Table S7: Number of amino acid residue in CDR3**

| **Number of Amino acid residue** | **Ig V_H_** | | |  | **Ig V_LK_** | | |
| --- | --- | --- | --- | --- | --- | --- | --- |
|  | **Anti-phOx** | **Anti-SCoVN** | |  | **Anti-phOx** | | **Anti-SCoVN** |
| 8 | 2 |  | |  | 1 | |  |
| 9 | 9 |  | |  | 8 | | 4 |
| 10 | 1 | 1 | |  |  | |  |
| 11 | 2 |  | |  |  | |  |
| 12 |  | 1 | |  |  | |  |
| 13 |  |  | |  |  | |  |
| 14 |  |  | |  | 2 | |  |
| 15 |  |  | |  |  | |  |
| 16 |  |  | |  | 1 | |  |
| 17 | 2 |  | |  | 3 | | 1 |
| 18 | 1 | 1 | |  | 5 | | 1 |
| 19 |  |  | |  |  | |  |
| 20 |  |  | |  |  | |  |
| 21 | 1 | 1 | |  |  | |  |
| 22 | 2 |  | |  |  | |  |
| 23 |  | 2 | |  |  | |  |
| **Total scFv clone analyzed** | **20** | | **6** |  | **20** | **6** | |

* Putative length of CDR3 is based on sequence analysis by VBASE2, and the data are extracted from Supplementary Tables 2, 4 and 5.
